# 720. The clinical outcomes of hypervirulent carbapenem-resistant *Klebsiella pneumoniae* (CRKP) infection were not poorer than that of classical CRKP infection

**DOI:** 10.1093/ofid/ofad500.782

**Published:** 2023-11-27

**Authors:** Ya Hu, Zhiyong Zong

**Affiliations:** West China Hospital, Chengdu, Sichuan, China; West China Hospital, Chengdu, Sichuan, China

## Abstract

**Background:**

Hypervirulent carbapenem-resistant *Klebsiella pneumoniae* (hv-CRKP) was superbug for patients. Despite the high resistance and virulence, the clinical outcomes of hv-CRKP induced infections were not ever measured before.

**Methods:**

We conducted a retrospective observational study of patients hospitalized at a comprehensive teaching hospital from July 2019 to June 2022 with CRKP being detected from their clinical samples. Isolates presenting resistance to meropenem and carrying carbapenemase-producing genes were deemed as CRKP. Hypervirulence was defined by the existence of *iuc* and/or either *rmpA* or *rmpA2*. Hv-CRKP met both conditions of CRKP and hypervirulence, and cCRKP met only the condition of CRKP but not hypervirulence. The corresponding patients were subsequently aligned into hv-CRKP group and cCRKP group, respectively (Figure 1). Outcome variables were compared between hv-CRKP group and classical CRKP (cCRKP) group.

**Figure 1.**
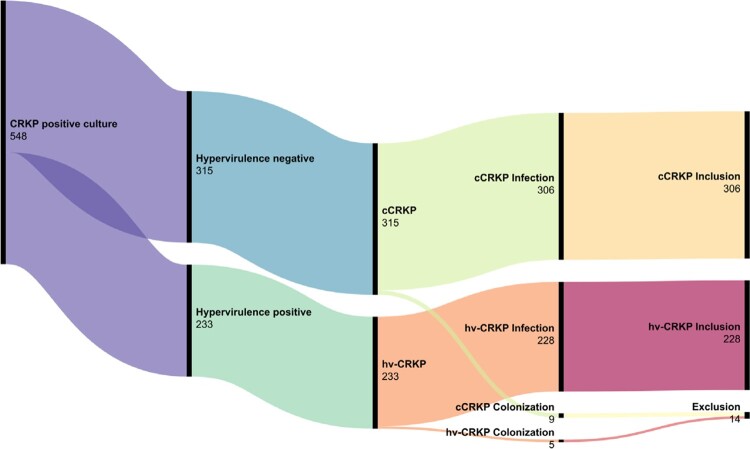
Screening workflow

**Results:**

In total, 534 patients were identified and included in this study, wherein 42.7% were of hv-CRKP infection and 57.3% were of cCRKP infection (Table 1). The 30-day all-cause mortality was 32.4% overall. The 30-day all-cause mortality of cCRKP group was a bit higher than hv-CRKP group (33.7% vs. 30.7%, p=0.513). Within BSI group, the hv-CRKP group presented higher 30-day all-cause mortality than cCRKP group (8.8% vs.7.5%, p=0.792). The 30-day infection attributable mortality was 79.8%. The length of stay (LOS) of CRKP infection patients ranged from 0 day to 1318 days with a median of 34 days. Only a half day difference in the mean LOS was seen between hv-CRKP group and cCRKP group (p=0.769). The mean post-culture LOS of CRKP infection was 14 days ranging from 0 to 1154. Comparing to cCRKP group, hv-CRKP group had a two-day shorter median of post-culture LOS (p=0.244). The survival of CRKP infection was 122 days in median and ranged from 0 to 1260 days. Hv-CRKP group had a diminished mean survival as to cCRKP group (18 days vs. 25 days, p=0.167). Survival curve presented a similar effect of both groups (Figure 2, p=0.926).

Table 1-1

**Figure:**
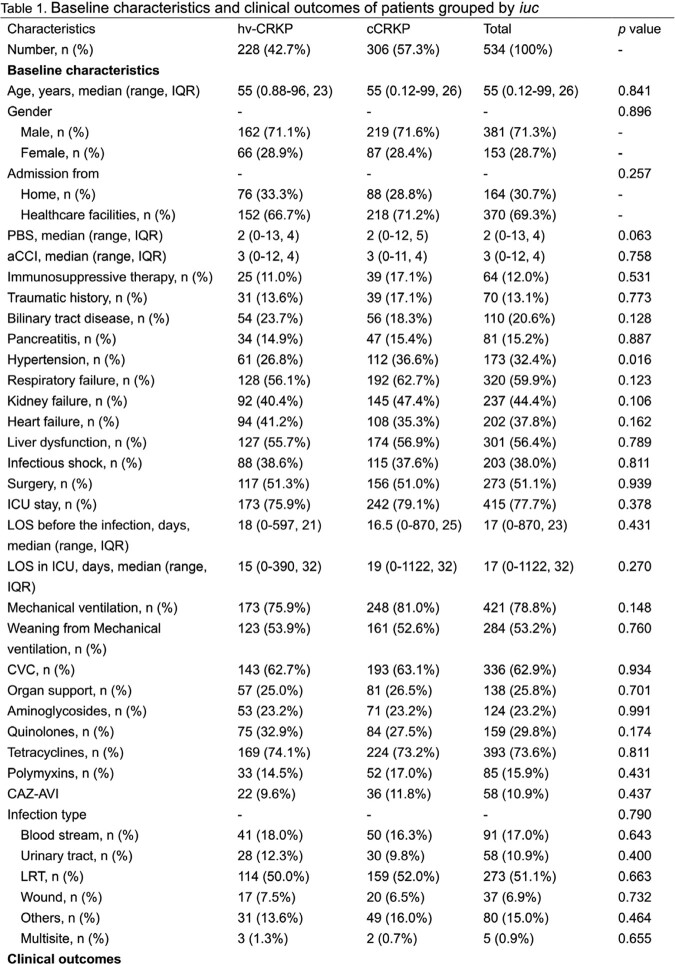

Table 1-1 Table 1-2

**Figure:**
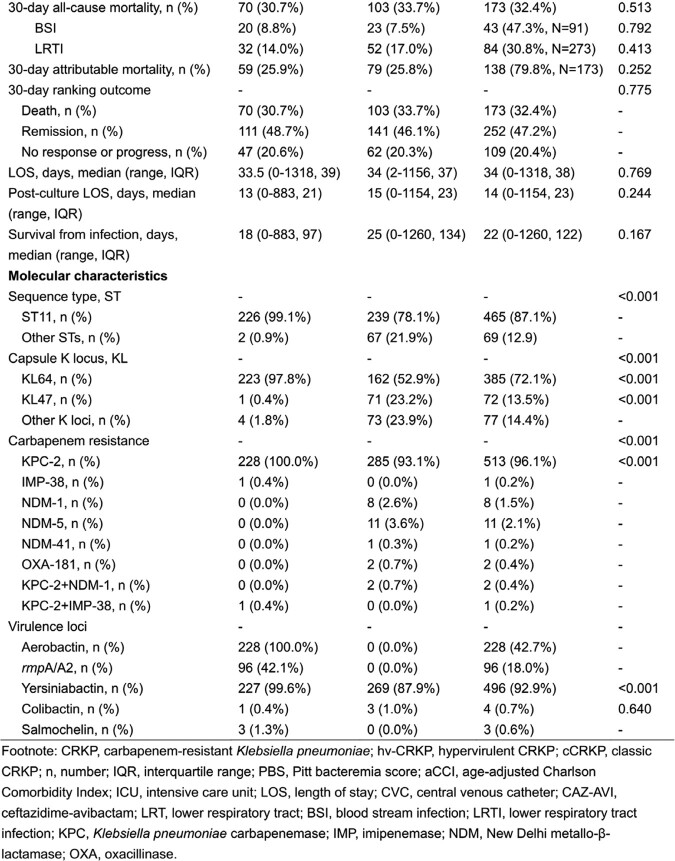

Table 1-2

**Figure 2.**
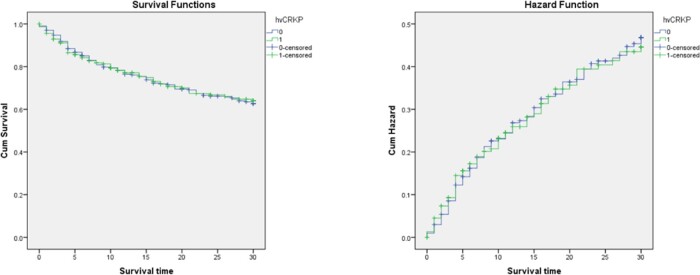
Kaplan-Meier curve for 30-day mortality

**Conclusion:**

Patients with hv-CRKP and patients with cCRKP had similar clinical outcomes.The clinical outcomes of hv-CRKP infection were not poorer than that of cCRKP infection.

**Disclosures:**

**All Authors**: No reported disclosures

